# Interhospital referral of colorectal cancer patients: a Dutch population-based study

**DOI:** 10.1007/s00384-021-03881-2

**Published:** 2021-03-20

**Authors:** A. K. Warps, M. P. M. de Neree tot Babberich, E. Dekker, M. W. J. M. Wouters, J. W. T. Dekker, R. A. E. M. Tollenaar, P. J. Tanis, On behalf of the Dutch ColoRectal Audit

**Affiliations:** 1 Department of Surgery and Biomedical Data Sciences, Leiden University Medical Center, Albinusdreef 2, 2333ZA Leiden, Netherlands; 2 Scientific Bureau, Dutch Institute for Clinical Auditing, Rijnsburgerweg 10, 2333AA Leiden, Netherlands; 3 Department of Gastroenterology and Hepatology, Amsterdam University Medical Centers, University of Amsterdam, Meibergdreef 9, 1105AZ Amsterdam, Netherlands; 4 Department of Surgical Oncology, Antoni van Leeuwenhoek Hospital, Plesmanlaan 121, 1066CX Amsterdam, Netherlands; 5 Department of Surgery, Reinier de Graaf Groep, Reinier de Graafweg 5, 2625AD Delft, Netherlands; 6 Department of Surgery, Cancer Centre Amsterdam, Amsterdam University Medical Centers, University of Amsterdam, Meibergdreef 9, 1105AZ Amsterdam, Netherlands

**Keywords:** Interhospital referral, Centralization, Waiting time, Colorectal cancer care

## Abstract

**Purpose:**

Interhospital referral is a consequence of centralization of complex oncological care but might negatively impact waiting time, a quality indicator in the Netherlands. This study aims to evaluate characteristics and waiting times of patients with primary colorectal cancer who are referred between hospitals.

**Methods:**

Data were extracted from the Dutch ColoRectal Audit (2015-2019). Waiting time between first tumor-positive biopsy until first treatment was compared between subgroups stratified for referral status, disease stage, and type of hospital.

**Results:**

In total, 46,561 patients were included. Patients treated for colon or rectal cancer in secondary care hospitals were referred in 12.2% and 14.7%, respectively. In tertiary care hospitals, corresponding referral rates were 43.8% and 66.4%. Referred patients in tertiary care hospitals were younger, but had a more advanced disease stage, and underwent more often multivisceral resection and simultaneous metastasectomy than non-referred patients in secondary care hospitals (*p*<0.001). Referred patients were more often treated within national quality standards for waiting time compared to non-referred patients (*p*<0.001). For referred patients, longer waiting times prior to MDT were observed compared to non-referred patients within each hospital type, although most time was spent post-MDT.

**Conclusion:**

A large proportion of colorectal cancer patients that are treated in tertiary care hospitals are referred from another hospital but mostly treated within standards for waiting time. These patients are younger but often have a more advanced disease. This suggests that these patients are willing to travel more but also reflects successful centralization of complex oncological patients in the Netherlands.

**Supplementary Information:**

The online version contains supplementary material available at 10.1007/s00384-021-03881-2.

## Introduction

Waiting times in oncologic care may cause psychological harm to patients [1–5]. The underlying concern relates to potential progression of the tumor during the diagnostic work-up and waiting time until start of first treatment [6–9]. In the Netherlands, this has led to the development of quality standards for waiting times, set up by the Dutch Federation of Oncologic Specialties (*SONCOS)* [10]. For colorectal cancer, a maximum waiting time from diagnosis until first treatment of 5 weeks was established. If a patient is referred to another hospital, an extra 3 weeks can be added to the maximum waiting time. Although no profound scientific evidence exists for the association of delays in colorectal cancer treatment on survival [11–15], which could be due to the slow tumor progression [16, 17], it is increasingly seen as a hospital quality indicator.

Waiting times remain a challenge for hospitals [18], and the ongoing centralization of complex surgery to high-volume centers [19–21], causes an increasing burden on hospital resources [19, 22]. Although most of the waiting time seems to take place in the (pre-) diagnostic phase [23–25], time from diagnosis until treatment has the most impact on patient satisfaction [25].

In the Dutch healthcare system, cancer networks organize weekly consultation of regional hospitals by a tertiary hospital. Minimal hospital volumes and defining complexity of disease that needs referral to an expert center are the driving forces in interhospital referral. There is, however, minimal insight in redistribution of patients through interhospital referral and the impact on waiting times and subsequent outcomes. Therefore, this study aimed to determine the characteristics of patients referred between hospitals in the Netherlands, with the corresponding waiting times compared to patients who were not referred. For this purpose, hospitals were stratified as secondary and tertiary care hospitals.

## Methods

Data were derived from the Dutch ColoRectal Audit (DCRA), a disease-specific national audit. This audit is mandatory and registers patients, tumor, treatment, and short-term outcome characteristics of all patients undergoing resection for primary colorectal cancer in the Netherlands [26]. Referral status was included in the audit since January 2015.

Hospitals were categorized into secondary or tertiary care hospitals, based on specialization in the treatment of colorectal cancer and the availability of advanced treatment modalities. Of the 75 hospitals in the Netherlands in 2015-2019, eight university hospitals, one cancer center, and one referral center for locally advanced cancer were classified as tertiary care hospitals. Further information about the distribution and characteristics of Dutch hospitals have been described earlier [27].

### Patients

For this study, no ethical approval or informed consent was required under Dutch law. All patients (*n*=52,666) who underwent resection between January 1, 2015, and December 31, 2019, were evaluated. Minimal data requirements were tumor location, date of surgery, and 30-day/in-hospital mortality. In addition, only patients operated in an elective setting without synchronous tumors (*n*=46,572), and with information on the date of first tumor-positive biopsy and date of operation were selected, resulting in a total eligible number of 46,561 patients.

### Data extraction and outcome parameters

The following data were extracted from the DCRA database: patient and disease characteristics, procedural characteristics, and 30-day or in-hospital postoperative outcome.

For the evaluation of waiting times, the following dates were used from the DCRA: first tumor-positive biopsy, first multidisciplinary team meeting (MDT), and first treatment given to the patient (neoadjuvant (preoperative radiotherapy or systemic treatment), metastasectomy, or resection of the primary tumor).

Because of its potential implications for waiting times in secondary and tertiary care hospitals, patients were categorized into three groups: group (1) non-locally advanced/non-metastatic colon or rectal cancer (cT1-3N0-2M0); group (2) locally advanced/non-metastatic (cT4N0-2M0); and group (3) metastatic colon or rectal cancer (M1). If clinical stage was missing and no neoadjuvant treatment was given, pathological stage was used.

To assess possible delay in the diagnostic phase, time between first positive biopsy and MDT was calculated and categorized as 0–2, 3–4, 5–6, and ≥7 weeks. The date of the MDT was not registered in 2018, so only patients that underwent surgery between 2015 and 2017 or in 2019 were included for this specific analysis. To evaluate total waiting time, time from first tumor-positive biopsy until first treatment was calculated and categorized as ≤5 weeks, 6 to 8 weeks, and > 8 weeks, according to Dutch quality standards.

### Data analysis

Analyses were performed separately for colon and rectal cancer and for secondary and tertiary care hospitals. Patients were classified as non-referred (NRF) and referred (RF) based on registered referral status in the DCRA. Risk factors for a waiting time from first tumor-positive biopsy until treatment of more than 5 weeks were determined using multivariable logistic analysis. Multicollinearity was tested with the variance inflation factor (VIF). A VIF of >2.5 was considered as multicollinearity and resulted in removing one of the factors. Differences in waiting times were analyzed with a Kruskal-Wallis rank-sum test for continuous variables or Chi-square test for categorical variables. A *p*-value of less than 0.05 was considered statistically significant. All analyses were performed in R Studio version 3.6.1 (2020).

## Results

### Baseline characteristics and neoadjuvant treatment

A total of 31,560 patients with colon cancer and 15,001 with rectal cancer were included for analysis**.** Table 1 shows the characteristics of the NRF and RF patients per type of hospital (secondary and tertiary) for colon and rectum separately. In secondary care hospitals, 12.2% of the patients with colon cancer and 14.7% of the patients with rectal cancer were referred from another hospital. In tertiary care hospitals, this was 43.8% and 66.4%, respectively. Similar baseline differences between RF patients in tertiary care hospitals to NRF patients in secondary care hospitals were found for colon cancer and rectal cancer. RF patients were less often ≥75 years, had less often an ASA 3+ score, had less frequently a BMI of 30+, had a higher T-stage, had more often metastasis (M1), had undergone more often a surgical intervention preceding the resection, and received more often neoadjuvant treatment. In addition, RF rectal cancer patients in tertiary care hospitals had more often a tumor located ≤ 5 cm of the anal verge.

**Table 1:** Baseline characteristics

		Colon					Rectum				
		Secondary care (*N*=28,788)		Tertiary care (*N*=2772)			Secondary care (*N*=12,783)		Tertiary care (*N*=2218)		
		NRF	RF	NRF	RF	*p*^*a*^	NRF	RF	NRF	RF	*p*^*a*^
	Total *N* (%)	25,555 (88.8)	3233 (12.2)	1558 (56.2)	1214 (43.8)	*Secondary NRF vs. tertiary RF*	10,899 (85.3)	1884 (14.7)	745 (33.6)	1473 (66.4)	*Secondary NRF vs. tertiary RF*
Patient		*n* (%)	*n* (%)	*n* (%)	*n* (%)		*n* (%)	*n* (%)	*n* (%)	*n* (%)	
Age (yrs)	≥75	8663 (33.9)	642 (19.9)	460 (29.5)	217 (17.9)	*p*<0.001	2700 (24.8)	337 (17.9)	137 (18.4)	198 (13.4)	*p*<0.001
Gender	Male	13,441 (52.6)	1789 (55.3)	860 (55.2)	632 (52.1)	*p*=0.734	6875 (63.1)	1,219 (64.7)	278 (37.3)	559 (37.9)	*p*=0.452
ASA score	III+	7227 (28.3)	614 (19.0)	493 (31.6)	243 (20.0)	*P*<0.001	2303 (21.1)	316 (16.8)	161 (21.6)	227 (15.4)	*P*<0.001
Charlson comorbidity index	3+	3506 (13.7)	293 (9.1)	316 (20.3)	156 (12.9)	*p*=0.413	1063 (9.8)	141 (7.5)	88 (11.8)	134 (9.1)	*p*=0.452
BMI (kg/m2)	30+	5277 (20.9)	657 (20.5)	319 (20.7)	220 (18.4)	*p*=0.038	1985 (18.5)	355 (19.1)	122 (16.6)	210 (14.4)	*P*<0.001
Previous abdominal surgery	Yes	287 (1.1)	35 (1.1)	37 (2.4)	15 (1.2)	*p*=0.823	91 (0.8)	20 (1.1)	15 (2.0)	19 (1.3)	*p*=0.110
Tumor											
Location of tumor	Ascending colon (incl. hepatic flexure)	11,853 (46.4)	1260 (39.0)	730 (46.9)	533 (43.9)	*p*=0.002					
	Transverse colon (incl. splenic flexure)	2742 (10.7)	344 (10.6)	182 (11.7)	146 (12.0)						
	Descending colon	1633 (6.4)	236 (7.3)	91 (5.8)	52 (4.3)						
	Sigmoid colon	9327 (36.5)	1393 (43.1)	555 (35.6)	483 (39.8)						
Distance from anal verge	≤5 cm						3990 (36.6)	756 (40.1)	327 (43.9)	761 (51.7)	*p*<0.001
	6–10 cm						3709 (34.0)	580 (30.8)	236 (31.7)	441 (29.9)	
	>10 cm						2649 (24.3)	435 (23.1)	160 (21.5)	230 (15.6)	
Tumor group	T1-3N0-2M0	21,638 (84.7)	2782 (86.1)	1202 (77.2)	585 (48.2)	*p*<0.001	9648 (88.6)	1658 (88.4)	579 (77.7)	737 (50.0)	*p*<0.001
	T4N0-2M0	2063 (8.1)	148 (4.6)	172 (11.0)	193 (15.9)		634 (5.8)	105 (5.6)	88 (11.8)	404 (27.4)	
	M1	1730 (6.8)	274 (8.5)	182 (11.7)	435 (35.8)		604 (5.5)	112 (6.0)	78 (10.5)	332 (22.5)	
Neo-adjuvant treatment											
Surgical intervention preceding the resection	No	24,357 (95.3)	3092 (95.6)	1485 (98.5)	1165 (96.0)	*p*<0.001	10,028 (92.0)	1736 (92.1)	654 (87.8)	1,105 (75.0)	*p*<0.001
	Stoma	455 (1.8)	24 (0.7)	26 (1.7)	117 (9.6)		556 (5.1)	65 (3.5)	58 (7.8)	303 (20.6)	
	Other (stent/metastasectomy/RFA/other)	743 (2.9)	117 (3.6)	47 (3.0)	106 (8.7)		315 (2.9)	83 (4.4)	33 (4.4)	65 (4.4)	
Neoadjuvant chemotherapy	Yes	341 (1.3)	58 (1.8)	58 (3.7)	247 (20.3)	*p*<0.001	1740 (16.0)	296 (15.7)	202 (27.1)	584 (39.6)	*p*<0.001
Neoadjuvant radiotherapy	No						5379 (49.4)	1032 (54.8)	315 (42.3)	355 (24.1)	*p*<0.001
	SCRT						2495 (22.9)	393 (20.9)	174 (23.4)	279 (18.9)	
	CRT						2856 (26.2)	438 (23.2)	244 (32.8)	825 (56.0)	
	Other, RTx scheme						124 (1.1)	16 (0.8)	12 (1.6)	12 (0.8)	

*NRF* not referred, *RF* referred. ^a^Chi-square test was used for all categorical variables. *RFA* Radiofrequency ablation. *SCRT *short course radiotherapy. *CRT* chemoradiotherapy. Missing <10% are not shown in table. ^a^Chi-square test

### Patient, tumor, and neoadjuvant treatment characteristics associated with referral to tertiary care hospitals

In colon cancer patients, a significant higher odds for referral to a tertiary care hospital was found for locally advanced and metastatic disease (AOR 2.514, 95% CI 2.074–3.046 for group 2 (T4N0-2M0) and AOR 5.560, 95% CI 4.740–6.522 for group 3 (M1)), if a stoma was created for obstruction (AOR 2.010, 95% CI 1.522–2.656) and when neo-adjuvant chemotherapy was given (AOR 6.231, 95% CI 4.989–7.783). Significant lower odds were observed for age 75+ (AOR 0.535, 95% CI 0.454–0.631), ASA III+ (AOR 0.726, 95% CI 0.615–0.856), and when the primary tumor was located in the descending colon (AOR 0.532, 95% CI 0.381–0.743) (Table 2).

**Table 2:** Factors related with referral

		Colon	Rectum
		Adjusted odds ratio (95% CI)	Adjusted odds ratio (95% CI)
Age	<75 years	Ref.	Ref.
	≥75 years	*0.535 (0.454–0.631)*	*0.516 (0.433–0.616)*
ASA score	I–II	Ref.	Ref.
	III+	*0.726 (0.615–0.856)*	*0.692 (0.581–0.824)*
BMI (kg/m2)	≤30	Ref.	Ref.
	>30	1.045 (0.886–1.232)	*0.811 (0.680–0.967)*
Previous abdominal surgery	No	Ref.	Ref.
	Yes	1.195 (0.621–2.299)	*2.374 (1.357–4.156)*
Location of tumor	Ascending colon (incl. hepatic flexure)	Ref.	
	Transverse colon (incl. splenic flexure)	1.099 (0.890–1.351)	
	Descending colon	0.532 (0.381–0.743)	
	Sigmoid colon	0.921 (0.797–1.066)	
Tumor distance from anal verge	>10cm		Ref.
	6–10cm		*1.242 (1.301–1.497)*
	≤5cm		*1.881 (1.578–2.244)*
Tumor group	1. (T1-3N0-2M0)	Ref.	Ref.
	2. (T4N0-2M0)	*2.514 (2.074–3.046)*	*5.839 (4.938–6.909)*
	3. (M1)	*5.560 (4.740–6.522)*	*5.071 (4.226–6.085)*
Surgical intervention preceding the resection	No	Ref.	Ref.
	Stoma	*2.010 (1.522–2.656)*	*1.968 (1.639–2.362)*
Neo adjuvant chemotherapy	No	Ref.	Ref.
	Yes	*6.231 (4.989–7.783)*	*1.458 (1.259–1.686)*
Neoadjuvant radiotherapy	No		Ref.
	Yes		*1.462 (1.248–1.712)*
Modelfit: Nagelkerke *R*^2^:		*0.167*	*0.222*

Multivariable logistic regression assessing patient, tumor, and neo-adjuvant factors associated with referral to tertiary hospitals compared to non-referred patient in secondary care hospitals

In rectal cancer, significant higher odds for referral to a tertiary hospital was found for previous abdominal surgery (AOR 2.374**,** 95% CI 1.357–4.156**),** tumor location (≤5cm from anal verge; AOR 1.881, 95% CI 1.578–2.244; 6–10cm AOR 1.242, 95% CI 1.301–1.497), locally advanced and metastatic tumors (AOR 5.839, 95% CI 4.938–6.909 for group 2 and AOR 5.071, 95% CI 4.226–6.085 for group 3), construction of a stoma preceding the resection (AOR 1.968, 95% CI 1.639–2.362), and neoadjuvant therapy (AOR 1.458, 95% CI 1.259–1.686 for chemotherapy and AOR 1.462, 95% CI 1.248–1.712 for radiotherapy). In contrast, a significant lower odds ratio was seen for age 75+ (AOR 0.516, 95% CI 0.433–0.616), ASA III + (AOR 0.692, 95% CI 0.680–0.967), and BMI 30+ (AOR 0.811, 95% CI 0.680–0.967).

### Waiting time

**Fig. 1:**
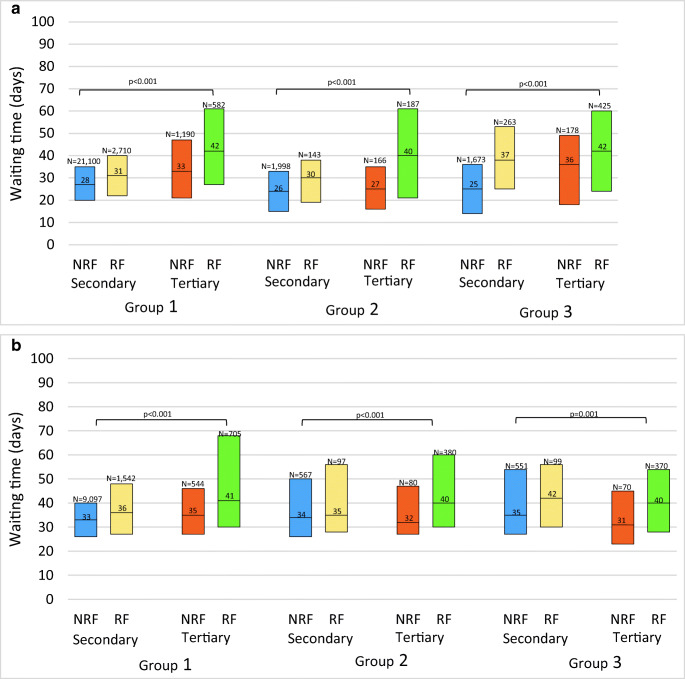
**a** Waiting times in days for colon cancer patients. Figure 1 a shows the waiting time (days) for NRF and RF colon cancer patients in secondary and tertiary hospitals. A Chi-square test was used. Group 1, T1-3N0-2M0; group 2, T4N0-2M0; group3, M1. **b** Waiting times in days for rectal cancer patients. Figure 1 b shows the waiting time (days) for NRF and RF rectal cancer patients in secondary and tertiary hospitals. A Chi-square test was used. Group 1, T1-3N0-2M0; group 2, T4N0-2M0; group3, M1

After excluding unknown T or M stage and inconclusive endoscopic biopsy, a total of 30,615 patients with colon cancer and 14,039 with rectal cancer were evaluable for analyzing waiting times. Figure 1 shows the median waiting times, with their corresponding interquartile range (IQR) from first tumor-positive biopsy until first treatment for secondary and tertiary care hospitals, stratified for the different patient groups.

Within each group, all RF colon and rectal cancer patients in tertiary care hospitals had a significantly longer waiting time compared to NRF patients in secondary care hospitals. Mean waiting time differences ranged from 5 days (group 3 rectal cancer, 40 vs. 35 days, *p*<0.001) to 17 days (group 3 colon cancer, 42 vs. 25 days, *p*<0.001).

Within secondary care hospitals, waiting time was longer for RF compared to NRF patients in all colon cancer subgroups, with waiting time differences ranging from 3 (group 1, 31 vs. 28 days, *p*<0.001) to 12 days (group 3, 37 vs. 25 days, *p*=0.001). Referred patients that were treated in secondary care hospitals waited longer than NRF patients in case of non-locally advanced/non-metastasized disease (group 1, 36 vs. 33 days, *p*<0.001) and metastasized disease (group 3, 42 vs. 35 days, *p*=0.003).

Among all colon cancer subgroups in tertiary care hospitals, RF patients waited significantly longer than NRF patients, ranging from 6 (group 3, 42 vs. 36 days, *p*<0.001) to 13 days (group 2, 40 vs. 27 days, *p*<0.001). For rectal cancer treated in tertiary centers, these waiting time differences ranged from 6 (group 1, 41 vs. 35 days, *p*<0.001) to 9 days (group 3, 40 vs. 31 days, *p*=0.006).

### Adherence to quality standards

Supplementary table 1 shows that for colon cancer patients in secondary care hospitals, the overall waiting time was ≤5 weeks in 73.6% for NRF patients and ≤8 weeks in 89.2% for RF patients (*p*<0.001). Corresponding percentages in tertiary care hospitals were 57.1% and 70.4% (*p*<0.001), respectively.

For rectal cancer treatment in secondary care hospitals, 58.3% of the NRF patients were treated ≤5 weeks, and 82.7% of the RF patients were treated ≤8 weeks (*p*<0.001). In tertiary hospitals, these percentages were 53.9% and 76.3%, respectively (*p*<0.001). Supplementary figure 1 gives an overview of median waiting times per hospital between first tumor positive biopsy and first treatment for secondary and tertiary care hospitals.

### Diagnostic and referral delay

**Fig. 2:**
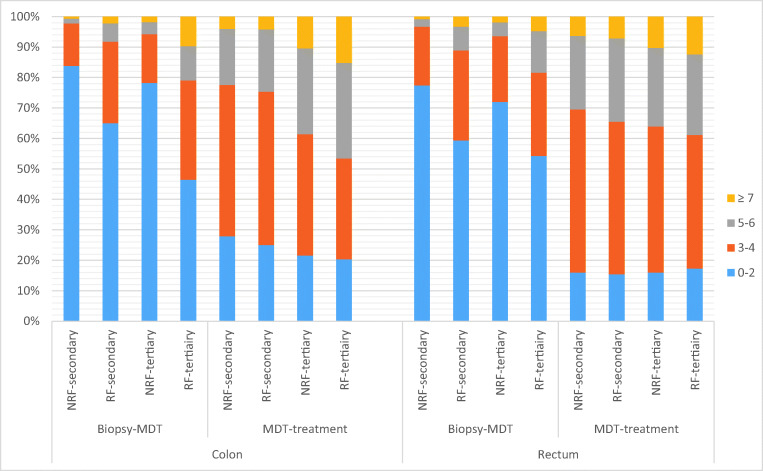
Waiting times for biopsy until MDT and MDT until treatment. Figure 2 shows the waiting times (weeks) for biopsy until MDT and MDT until treatment for NRF and RF patient in secondary and tertiary hospitals

The date of multidisciplinary team meeting (MDT) was registered in 20,229 colon and 9954 rectal cancer patients. Time from first tumor-positive biopsy until MDT was significantly longer for RF colon cancer patients compared to NRF patients in both secondary (*p*<0.001) and tertiary care hospitals (*p*<0.001) as shown in Fig. 2. This was also found for rectal cancer in both types of hospitals. The time from first MDT until first treatment was significantly longer for RF colon cancer patients compared to NRF patients, but with minimal absolute differences. In contrast, the time from first MDT until first treatment did not significantly differ between RF and NRF rectal cancer patients.

### Surgical characteristics

Table 3 shows that RF patients in tertiary care hospitals differed significantly for the majority of surgical characteristics compared with NRF patients in secondary hospitals. RF colon and rectal cancer patients in tertiary care hospitals had a higher risk of getting a stoma (*p*<0.001 and *p*<0.001, respectively), had more often an additional resection due to tumor invasion (*p*<0.001 and *p*<0.001, respectively), and had more often a simultaneous resection for metastasis (*p*<0.001 and *p*<0.001, respectively) compared to NRF patients in secondary hospitals.

**Table 3:** Surgical characteristics

		Colon					Rectum				
		Secondary care (*N*=28,788)		Tertiary care (*N*=2772)			Secondary care (*N*=12,783)		Tertiary care (*N*=2218)		
		NRF	RF	NRF	RF	*p*^*a*^	NRF	RF	NRF	RF	*p*^*a*^
	Total *N* (%)	25,555 (88.8)	3233 (12.2)	1558 (56.2)	1214 (33.8)	*Secondary NRF vs. tertiary RF*	10,899 (85.3)	1884 (14.7)	745 (33.6)	1473 (66.4)	*Secondary NRF vs. tertiary RF*
Surgery											
Stoma	No	24,114 (94.4)	3113 (96.3)	1423 (91.3)	1016 (83.7)	<0.001	3974 (36.5)	750 (39.8)	267 (35.8)	461 (31.3)	<0.001
	Loop ileostomy	228 (0.9)	38 (1.2)	24 (1.5)	38 (3.1)		2,394 (22.0)	346 (18.4)	137 (18.4)	238 (16.2)	
	End ileostomy	207 (0.8)	10 (0.3)	28 (1.8)	21 (1.7)		86 (0.8)	7 (0.4)	12 (1.6)	15 (1.0)	
	Loop colostomy	51 (0.2)	5 (0.2)	13 (0.8)	30 (2.5)		182 (1.7)	26 (1.4)	78 (10.5)	133 (9.0)	
	End colostomy	934 (3.7)	59 (1.8)	66 (4.2)	101 (8.3)		3,499 (32.1)	450 (23.9)	227 (30.5)	586 (39.8)	
	Stoma, not further specified	3 (0.0)	1 (0.0)	4 (0.3)	7 (0.6)		8 (0.1)	1 (0.1)	2 (0.3)	1 (1.0)	
Additional resection due to tumor invasion	Yes	563 (2.2)	60 (1.9)	83 (5.3)	233 (19.2)	<0.001	131 (1.2)	33 (1.8)	48 (6.4)	304 (20.6)	<0.001
Additional resection for metastasis	Yes	416 (1.6)	144 (4.5)	103 (6.6)	364 (30.0)	<0.001	99 (0.9)	45 (2.4)	45 (6.0)	217 (14.7)	<0.001

*NRF* not referred, *RF* referred. ^*a*^Chi-square test was used for all categorical variables. Missing<10% are not shown in table**.** ^*a*^Chi-square test

## Discussion

This population-based study shows that waiting times for referred colorectal cancer patients differ substantially from those of patients treated in their hospital of diagnosis. For both secondary and tertiary care hospitals, it is hard to meet the current Dutch waiting time standards in every single case, but remarkably this seems more difficult in NRF patients compared to RF patients. According to Dutch quality standards, waiting times for the latter patient population may be 3 weeks longer (eight versus five weeks), which explains this remarkable observation. A large proportion of patients treated in tertiary care hospitals consist of referred patients with advanced disease, both locally advanced as well as metastatic. The complexity of these patients also translates into the type of treatments given. For example, more diverting stomas were constructed in these patients with more often neoadjuvant treatment. From these observations, we conclude that centralization of more complex patients has been successful in the Netherlands over the past years, with efficient referral to tertiary care hospitals. For RF patients, longer waiting times mainly occur during diagnostic or referral phase (pre-MDT). Once patients have been discussed in the MDT, RF colon cancer patients experience only a minimal longer waiting time.

Waiting times are always of concern for patients and healthcare providers. Identifying and understanding factors associated with longer waiting times are essential information when discussing waiting time regulations. Simunovic et al. found that age was associated with treatment delay for breast, colon, lung, and prostate cancer [28]. Furthermore, Bilimoria et al. found that with several non-metastatic cancers (breast, colon, esophageal, gastric, liver, pancreatic, and rectal), besides age, also comorbidities (charslon2+), stage 1 disease (compared to stage 3), or treatment at a national cancer institute was associated with a treatment delay >30 days [18]. We found that RF patients had longer waiting times compared to NRF patients. We also found age, ASA III+, tumor stage, tumor location (part of the colon (colon cancer) or distance to the anal verge (rectal cancer)), and neoadjuvant treatment to be associated with RF. One might hypothesize that this reflects adequate prioritization by the surgeons. On the other hand, the RF population is a heterogeneous population that differs not only regarding tumor characteristics but also comprises patients with severe comorbidities that require specific perioperative care as well as relatively young, healthy, and highly educated patients that are more willing to travel for treatment. This demonstrates that centralization of more complex patients has been successful in the Netherlands over the past years, with efficient referral to tertiary care hospitals.

Nevertheless, we demonstrated that a substantial number of hospitals do not meet the waiting time standards and a significant impact of interhospital referral on waiting time was found. Centralization of complex care to tertiary care hospitals is important to pursue, but should ideally not cause delay in treatment. Tertiary care centers are not necessarily high-volume centers, and the majority of less complex care is probably best concentrated in high-volume secondary centers in an efficient manner. In the Netherlands, we are working on a potential solution and strategy to achieve this. By regionalization and echeloning of care with regional MDT meetings, waiting times can potentially be reduced with optimal centralization of complex care. Discussion of patients early in the cancer care process with good triage and exchange of patients between hospitals and willingness to refer are key elements. This will result in the referral of highly complex care to tertiary centers but also the exchange of low-complex patients to peripheral high-volume hospitals. By creating regional networks with a central portal where patients come in, efficient distribution of the “right patient to the right place” is aimed for.

Although no analysis of postoperative outcomes was performed, other studies did not find an association between delay in treatment and worse outcomes. Porter et al. showed that most of the delay was caused in the (pre) diagnostic phase. So, from the perspective of tumor biology, interventions should be aimed at improving access to these medical resources [25]. Bevis et al. found a significant delay until consultation by a specialist or initiation of treatment for patients referred through a non-fast track route, but this delay was not associated with a more advanced stage of disease or a reduction in potentially curative operations [13]. Also, the studies of Murchie et al. [29] and Helewa et al. [30] did not find a relationship between provider’s delay and stage at diagnosis or survival of colorectal cancer. In 2000, the UK introduced the 2-week rule to reduce diagnostic delay, which has not improved 2-year survival [31], and also meeting waiting time criteria showed no improvement in 1-year survival [32]. A systematic review that addressed the literature between 2008 and 2017 for treatment delay in colon cancer treatment found no association between delay and a reduced disease-free survival, 5-years survival, and overall survival for patients with colon cancer [33]. However, literature remains contradictory. For example, a systematic review of 34 studies (2000–2020) on 17 therapy indications by Hanna et al. found a consistent and significant increase in mortality for each 4 weeks delay of cancer surgery, with a hazard ratio of 1.06 (95% CI 1.01–1.12) for colectomy [34], and Lee showed that the risk of death was increased for patients with a waiting time of more than 30 days between diagnosis and treatment [35], whereas Strous et al. and Trepanier et al. showed that treatment exceeding a month did not decrease overall and recurrence-free survival [36, 37]. Amri et al. did not find worse cancer outcomes for treatment delay in colon cancer and even found an inverse relation between treatment delay and mortality and recurrence rates [38].

Even though most studies demonstrated that no (or at most a controversial) relationship between waiting time until treatment of colorectal cancer and worse outcomes exist, this should not be a reason to stop putting effort in reducing waiting times. It should be regarded as an indication that waiting times within reasonable ranges do not harm the patient from tumor biology perspective. However, psychological distress of the patients should also be taken into account. In a systematic review, Brocken et al. found that a rapid diagnostic pathway may be beneficial for the distress levels of patients eventually not having cancer, but this association was not seen in patients finally receiving the diagnosis of cancer [39]. In our study, the most significant differences in waiting time for RF compared to NRF patients were found in the time period before MDT. During this time interval, the additional diagnostic work-up and actual referral take place. Besides the fact that the referral process will take a certain amount of time, it is most likely that more complex patients also have a more extensive (diagnostic) work-up prior to the MDT, supporting the MDT in the discussion conducted for the most appropriate treatment options. To a certain extent, the additional diagnostic work-up and referral process run parallel to each other, making it challenging to quantify the actual proportion of waiting time that can be attributed solely to referral. For the pre-treatment phase, the need for a more comprehensive, personalized based approach may be beneficial, like Song et al. showed in a study on waiting times for the treatment of gastric and esophageal cancer [40]. Besides, extra waiting time could be a conscious choice, for example, to perform a more profound work-up, and does not necessarily have to be negative. Increased waiting time might also include patient delay of any reason (e.g., psychologically adapting to the diagnosis). Some patients have specific preferences for a hospital or surgeon. Like McConnell et al. discussed, the relationship between waiting times in colorectal cancer care and quality of care is complex [41], and multiple perspectives need to be taken into account.

The strength of the present study is the large number of patients and external validity related to the population-based data reflecting daily practice. However, some limitations need to be addressed. A certain degree of missing data is inevitable in population-based studies. Considering case-mix adjustment, there is always a possibility that not all contributing factors were included. Besides, we did not have any detailed information on patient, referral, or diagnostic delays (e.g., until colonoscopy). This prevents us from profoundly understanding the buildup of waiting times. Furthermore, no information on referral source (the type of hospital) was available. So, referral from secondary to tertiary care hospitals and the other way around could not be quantified or analyzed. Finally, the DCRA does not provide information on disease-free survival and overall (long-term) survival, which might be of relevance in the discussion on interhospital referral and waiting times.

In conclusion, this population-based study showed that a substantial number of hospitals do not meet current waiting time standards, and interhospital referrals had a significant impact on waiting time prior to the MDT. Referred colorectal cancer patients appeared to be younger healthier that have more advanced disease, in need of more complex treatment. The Dutch system seems to be able to efficiently concentrate low-volume high-complex disease. However, centralization of complex care seemed to be accompanied by an increased waiting time, and effort need to be made to prevent this since the impact of delay on survival is controversial. Regional networks, with efficient patient distribution based on the complexity of care needed, might prevent longer waiting times in tertiary care hospitals.

### Availability of data and material

The data that support the findings of this study are available from the Dutch Institute for Clinical Auditing (DICA) but are not publicly available. Data are however available from the authors upon reasonable request and with permission of the Dutch Institute for Clinical Auditing and the Dutch ColoRectal Audit Board.

### Code availability

Statistical codes that are used for this study is available from the authors upon reasonable request and with permission of the Dutch Institute

## Supplementary information

ESM 1 (DOCX 32 kb)
