# Supplementary material for: Interhospital referral of colorectal cancer patients: a Dutch population-based study
Source: Int J Colorectal Dis. 2021 Mar 20;36(7):1443–53. doi: 10.1007/s00384-021-03881-2 (PMC8195929; doi:10.1007/s00384-021-03881-2)
Supplement: Supplementary file 1 — (DOCX 32 kb) [file 384_2021_3881_MOESM1_ESM.docx]

**SUPPLEMENTARY FIGURE 1a: Median waiting times in days for non-referred patient in secondary and tertiary hospitals**

**SUPPLEMENTARY FIGURE 1b: Median waiting times in days for referred patient in secondary and tertiary hospitals**
